# Mayo Clinic experience with 1123 adults with acute myeloid leukemia

**DOI:** 10.1038/s41408-021-00435-1

**Published:** 2021-03-02

**Authors:** Kebede H. Begna, Walid Ali, Naseema Gangat, Michelle A. Elliott, Aref Al-Kali, Mark R. Litzow, C. Christopher Hook, Alexandra P. Wolanskyj-Spinner, William J. Hogan, Mrinal M. Patnaik, Animesh Pardanani, Darci L. Zblewski, Dong Chen, Rong He, David Viswanatha, Curtis A. Hanson, Rhett P. Ketterling, Ayalew Tefferi

**Affiliations:** 1 Division of Hematology, Department of Internal Medicine, Mayo Clinic, Rochester, MN USA; 2 Division of Hematopathology, Department of Internal Medicine, Mayo Clinic, Rochester, MN USA; 3 Division of Laboratory Genetics and Genomics, Department of Laboratory Medicine, Mayo Clinic, Rochester, MN USA

**Keywords:** Chemotherapy, Chemotherapy, Risk factors

## Abstract

Between 2004 and 2017, a total of 1123 adult patients (median age 65 years; 61% males) with newly diagnosed acute myeloid leukemia (AML), not including acute promyelocytic leukemia, were seen at the Mayo Clinic. Treatment included intensive (*n* = 766) or lower intensity (*n* = 144) chemotherapy or supportive care (*n* = 213), with respective median survivals of 22, 9, and 2 months (*p* < 0.01). Intensive chemotherapy resulted in complete remission (CR) and CR with incomplete count recovery (CRi) rates of 44 and 33%, respectively, with no difference in survival outcome between the two (*p* = 0.4). Allogeneic hematopoietic stem cell transplant (AHSCT) was documented in 259 patients and provided the best survival rate (median 55 months; *p* < 0.01). After a median follow-up of 13 months, 841 (75%) deaths were recorded. Multivariate analysis identified age >60 years (HR 2.2, 1.9–2.6), adverse karyotype (HR 2.9, 1.9–4.9), intermediate-risk karyotype (HR 1.6, 1.02–2.6), post-myeloproliferative neoplasm AML (HR 1.9, 1.5–2.4), and other secondary AML (HR 1.3 (1.1–1.6) as risk factors for shortened survival. These risk factors retained their significance after inclusion of *FLT3/NPM1* mutational status in 392 informative cases: *FLT3*+*NPM1*− (HR 2.8, 1.4–5.6), *FLT3*+/*NPM*+ (HR 2.6 (1.3–5.2), and *FLT3*−*NPM1*− (HR 1.8, 1.0–3.0).

## Introduction

Acute myeloid leukemia (AML) is a rapidly progressing clonal hematopoietic stem cell neoplasm that results in premature death and major comorbidity. The 2016 World Health Organization (WHO) classification system uses blood or bone marrow blast percentage and cytogenetic and molecular information in defining and classifying AML^1^. From a clinical perspective, AML is subcategorized into primary (de novo) and secondary. The latter includes AML arising from antecedent myeloid malignancies, such as myelodysplastic syndromes (MDS), myeloproliferative neoplasm (MPN), MDS/MPN overlap, and therapy-related AML.

Current diagnosis of AML requires the presence of ≥20% myeloid blasts circulating or in the bone marrow; in addition, diagnosis of AML is implied in the presence of certain recurrent cytogenetic abnormalities, such as t(8;21)(q22;q22.1)(*RUNX1-RUNX1T1)*, inv(16)(p13.1q22) or t(16;16)(p13.1;q22)(*CBFB-MYH11)*, and t(15;17)(q22;q12)*(PML/RARA)*, regardless of the blast percentage^1,2^. The diagnosis, prognostication, treatment, and monitoring of acute promyelocytic leukemia (APL) is uniquely different from other AML subtypes.

Treatment of non-APL AML has not changed, in a substantial manner, in the past four decades except the recent addition of some targeted therapies with uncertain long-term benefit. The “standard” involves combination chemotherapy (3 once-daily injections of anthracycline with continuous 7-day infusion of cytarabine, labeled as “3 + 7”) as induction with the goal of achieving complete remission (CR)^3,4^. Until recently, unless considered for clinical trial involving targeted therapies, or limited by patient’s age or co-morbid conditions, one does not need to know the cytogenetic (except in cases with core binding factor AML where early addition of Gemtuzumab with induction may have additional value) or molecular profile to start induction therapy in non-APL AML, even in *FLT3*-ITD-positive cases where midostaurin is started on day 8^5^. Long-term disease-free survival in AML is possible only with consolidation therapy. This may be in the form of several cycles of chemotherapy or allogeneic hematopoietic stem cell transplant (AHSCT) in the favorable and adverse risk groups, respectively.

In the current study, we share our 14 years’ real-life experience in 1123 consecutive and cytogenetically annotated adult AML patients seen at the Mayo Clinic. Our objectives were (1) to describe clinical and laboratory features and treatment outcome and real-life experience of non-APL AML in a large cohort of unselected adult patients seen at a single institution and (2) to examine the application of previously established risk factors in our study population.

## Patients and methods

The current study was approved by the Mayo Clinic institutional review board. One thousand one hundred and twenty-three consecutive and cytogenetically annotated patients with the diagnosis of non-APL AML were identified from the Mayo Clinic AML database and the study period spanned from January 1, 2004 through December 31, 2017. Study eligibility criteria included age ≥18 years, diagnosis of AML based on 2016 WHO criteria, and availability of cytogenetic data. The following AML subcategories were included: AML with recurrent cytogenetic abnormalities, AML with MDS-related changes, therapy-related AML, AML following MPN, AML-*NOS*, myeloid sarcoma, and AML of ambiguous linage. Patients were subcategorized into primary (de novo) and secondary AML; the latter was further subdivided into AML with MDS-related changes, AML following MPN, chronic myelomonocytic leukemia (CMML) or MDS/MPN, and therapy-related AML. In all instances, bone marrow examination and cytogenetic studies were performed or reviewed at the Mayo Clinic. Cytogenetic analysis and reporting of results was done according to the International System for Human Cytogenetic Nomenclature criteria^6–9^. Cytogenetic risk stratification into favorable, intermediate, and adverse groups was based on the 2017 European LeukemiaNet (ELN) criteria^2^. Whenever available, we have added the molecular profile to this risk stratification. Commonly available molecular information included *FLT3*-ITD, *NPM1*, and *CEBPA* mutational status. Favorable cytogenetic risk category included t(8;21)(q22;q22.1), *RUNX1-RUNX1T1*; inv(16)(p13.1q22) or t(16;16)(p13.1;q22), *(CBFB-MYH11)*. Adverse cytogenetic risk category included t(6;9)(p23;q34.1)*DEK-NUP214*; t(v;11q23.3);*KMT2A rearranged*; t(9;22)(q34.1;q11.2);*BCR/ABL1*; inv(3)(q21.3q26.2) *or* t(3;3)(q21.3;q26.2); *GATA2,EV1 (or MECOM)*; −5 or del(5q); −7; −17/abn(17p); and complex karyotype. Intermediate cytogenetic risk included normal karyotype and abnormal karyotype not included the in favorable or adverse groups. Follow-up information was updated as of May 2018 by reviewing charts, telephone calls to patients and their local hematologist, and using social security death index as necessary. For patients with a diagnosis of therapy-related AML, individual patient information was reviewed meticulously about their diagnosis and exposure to chemotherapy and/or radiation.

Type of therapy included induction (or intensive) chemotherapy, less intensive treatment such as with hypomethylating agents (HMA), and supportive care alone. Information with regard to marrow recovery was collected to evaluate CR according to International Working Group recommendation^10^. CR was defined as normal bone marrow morphology with <5% blast, absolute neutrophil count >1 × 10^9^/L, and platelet count >100 × 10^9^/L. CR with incomplete blood count recovery (CRi) met all the criteria for CR with the exception of either platelet or neutrophil count recovery; patients not categorized as CR or CRi were classified as “no remission”.

Statistical analysis considered clinical and laboratory variables at the time of diagnosis and as necessary at the time of follow-up or remission. Median (range) and frequencies (percentage) were used for continuous and categorical variables. Those variables were compared based on the time of diagnosis (2004–2010 vs 2011–2017), among the subtypes of AML, and the two main subgroups (primary vs secondary). Categorical variables were compared using chi-square and considered significant for *p* value <0.05. Overall survival was calculated from the date of diagnosis to death regardless of cause, and patients who were alive were censored at last follow-up. Patients who underwent AHSCT were censored at the date of transplant. Relapse-free survival was calculated from the date of reported CR to documented peripheral or bone marrow relapse with blast percentage of ≥5%; patients without relapse were censored at the date of last follow-up. Overall and relapse-free survival curves were prepared using Kaplan–Meier method and compared using log-rank test. Multivariate Cox-regression analyses were used on pretreatment variables to identify significant risk factors. The JMP® Pro 13.0.0 software (SAS Institute, Cary, NC, USA) was used for all calculations.

## Results

### Patients

A total of 1123 consecutive patients (median age 65 years, range 18–94; 61% males) with AML met the inclusion criteria; the clinical and laboratory characteristics of the study patients are outlined in Table 1. Forty percent (*n* = 449) of patients were seen from January 2004 to December 2010, and the rest after January 2011 (*n* = 674); more patients with secondary AML (48%) were diagnosed in the second half of the study period vs the first half (39%), and more patients (17%) received HMA in the second half of the study period vs the first half (7%), which might have been related to the approval and availability of HMAs.

**Table 1:** Clinical and laboratory characteristics of 1123 acute myeloid leukemia (non-APL) patients.

Variable	All patients, *n* = 1123 (100%)
Age (years), median (range)	65 (18–94)
Age groups	
<60 years	404 (36 %)
≥60 years	719 (64%)
Gender	
Male	689 (61%)
Female	434 (39%)
Year of diagnosis	
2004–2010	449 (40%)
2011–2017	674 (60%)
Leukocyte ×10^9^/L median (range)	6.5 (0.4–350)
Platelets ×10^9^/L median (range)	58 (3–1550)
Peripheral blood blast, % median (range)	19 (0–98)
Bone marrow blast, % median (range)	47 (0–99)
AML subtypes (practical classification)	
Primary (de novo)	626 (56%)
Secondary (post-myeloid malignancy)	388 (34%)
Therapy related	109 (10%)
AML subtype:	
Primary (de novo)	626 (56%)
Secondary (post-myeloid and therapy related)	497 (44%)
European LeukemiaNet	
Favorable	47 (4%)
Intermediate	650 (58%)
Adverse	426 (38%)
Molecular (genetic) findings (*n* = 392)	
FLT3-ITD and NPM1 negative	256 (65%)
FLT3-ITD negative and NPM1 positive	67 (17%)
FLT3-ITD and NPM1 positive	39 (10%)
FLT3-ITD positive and NPM1 negative	30 (8%)
Type of therapy	
Aggressive induction	766 (68%)
Hypomethylating agents	144 (13%)
No therapy	213 (19%)
Induction therapy outcome (*n* = 760)	
Complete remission (CR)	331 (44%)
CR with incomplete hematologic recovery (CRi)	248 (33%)
No remission	180 (23%)
Stem cell transplant	
Yes	256 (34%)
No	506 (66%)
Deaths	841 (75%)

AML was primary in 626 (56%) patients and secondary in 497 (44%); the latter included 388 patients with antecedent myeloid neoplasm and 109 therapy-related AML. Comparison of clinical, laboratory, and outcome measurements between primary and secondary AML are outlined in Table 2. Cytogenetic risk distribution, according to ELN classification, was favorable in 47 (4%), intermediate in 650 (58%), and adverse in 426 (38%) patients. The lower percentage of patients with favorable risk is attributable to exclusion of APL cases in the current study. There was no significant difference in the distribution of cytogenetic risk groups during the two study periods.

**Table 2:** Clinical and laboratory characteristics of 626 primary (de novo) and 497 secondary (post-myeloid and therapy related) acute myeloid leukemia (non-APL) patients with complete cytogenetic findings.

Variable	Primary (de novo) AML, *n* = 626	Secondary (post-myeloid and therapy related) AML, *n* = 497	*p* value
Age (years), median (range)	62 (18.7–92)	67 (18–94)	<0.0001
Age groups			
<60 years	275 (44)	129 (26)	**<0.0001**
≥60 years	351 (56)	368 (74)	
Gender			
Male	364 (58)	325 (65)	**0.01**
Female	262 (42)	172 (35)	
Year of diagnosis			
2004–2010	274 (44)	175 (35)	**0.004**
2011–2017	352 (56)	322 (65)	
Leukocyte ×10^9^/L median (range)	8.6 (0.3–350)	4.5 (0.1–246)	**<0.0001**
Platelets ×10^9^/L median (range)	64 (3–1550)	50 (2.6–1149)	**0.0002**
Peripheral blood blast, % median (range)	24 (0–131)	14 (0–99)	**<0.0001**
Bone marrow blast, % median (range)	60 (0–99)	34 (0–99)	**<0.0001**
European LeukemiaNet			
Favorable	39 (6)	8 (2)	**<0.0001**
Intermediate	419 (67)	231 (46)	
Adverse	168 (27)	258 (52)	
Molecular (genetic) findings (*n* = 392)			
FLT3-ITD and NPM1 negative	133 (56)	123 (80)	**<0.0001**
FLT3-ITD negative and NPM1 positive	47 (20)	20 (13)	
FLT3-ITD and NPM1 positive	33 (14)	6 (4)	
FLT3-ITD positive and NPM1 negative	25 (10)	5 (3)	
Type of therapy			
Intensive (induction) therapy	484 (77)	282 (57)	**<0.0001**
Hypomethylating agents	60 (10)	84 (17)	
No therapy	82 (13)	131 (26)	
Induction therapy outcome (*n* = 763)			
Complete remission (CR)	249 (52)	84 (30)	**<0.0001**
CR with incomplete hematologic recovery (CRi)	148 (31)	100 (35)	
No remission	84 (17)	98 (35)	
Stem cell transplant (*n* = 1118)			
Yes	163 (26)	98 (20)	**0.01**
No	460 (74)	397 (80)	
Deaths	418 (67)	397 (80)	**<0.0001**
Median survival, months (range)	27.9 (22–35)	9.2 (7.7–11)	**<0.0001**

Bold values indicates the variable to describe primary and secondary AML including their *p* values.

Patients with primary AML (*n* = 626, median age 62 years, range 18–92) were younger than those with either secondary (*n* = 388, median age 68 years, range 18–94) or therapy-related AML (*n* = 109, median age 65 years, range 19–90) (*p* < 0.01). As expected, more patients with adverse karyotype were seen in secondary (52%) and therapy-related (51%) vs primary (27%) AML (*p* < 0.01; Table 2). Induction chemotherapy was given to 78% of patients with primary, 57% of those with secondary, and 58% of patients with therapy-related AML; supportive care alone was more likely to be instituted in secondary (26%) and therapy related (27%) vs primary AML (13%; *p* < 0.01; Table 2). Table 3 summarizes clinical and laboratory features among patients receiving induction chemotherapy vs those who received either less intensive chemotherapy or supportive care alone.

**Table 3:** Clinical and laboratory characteristics of acute myeloid leukemia (Non-APL) patients with complete cytogenetic findings who received aggressive (Induction) (*n* = 766) vs less aggressive (*n* = 144 or no (*n* = 213) chemotherapy.

Variable	Intensive therapy, *n* = 766 (100%)	Less intensive, *n* = 144 (100%)	No therapy, *n* = 213 (100%)	*p* value
Age (years), median (range)	60 (18–88)	75 (47–92)	75 (25–94)	**<0.0001**
Age categories				
<60 years	376 (49)	5 (4)	23 (11)	<**0.0001**
≥60 years	390 (51)	139 (96)	190 (89)	
Gender				
Male	442 (58)	98 (68)	149 (70)	**0.0009**
Female	324 (42)	46 (32)	64 (30)	
Year of diagnosis				
2004–2010	319 (42)	31 (22)	99 (46)	0.01
2011–2017	447 (58)	113 (78)	114 (54)	
Leukocyte ×10^9^/L (median)	7.6 (0.1–350)	3.4 (0.4–194)	6.9 (0.3–246)	**0.003**
Platelet ×10^9^/L (median)	56 (2.6–943)	71 (7–1550)	54 (3–1149)	0.01
Peripheral blood blast, % (median)	22 (0–99)	8 (0–95)	18 (0–97)	**<0.0001**
Bone marrow blast, % (median)	53 (0–99)	35 (0–94)	38 (0–99)	**<0.0001**
AML subtypes (practical classification)				
Primary (de novo)	484 (63)	60 (42)	82 (38)	**<0.0001**
Secondary (post-myeloid malignancy)	219 (29)	67 (47)	102 (48)	
Therapy related	63 (8)	17 (11)	29 (14)	
AML subtype				
Primary (de novo)	484 (63)	60 (42)	82 (38)	**<0.0001**
Secondary (post-myeloid and therapy related)	282 (37)	84 (58)	131 (62)	
European LeukemiaNet				
Favorable	42 (5)	0 (0)	4 (2)	**<0.0001**
Intermediate	457 (60)	94 (65)	99 (46)	
Adverse	267 (35)	50 (35)	110 (52)	
Molecular (genetic) findings (*n* = 392)				
FLT3-ITD and NPM1 negative	175 (61)	41 (79)	40 (78)	**0.02**
FLT3-ITD negative and NPM1 positive	55 (19)	7 (13)	5 (10)	
FLT3-ITD and NPM1 positive	35 (12)	1 (2)	3 (6)	
FLT3-ITD positive and NPM1 negative	24 (8)	3 (6)	3 (6)	
Remission status (*n* = 763)				
Complete remission (CR)	331 (44)	2 (67)		0.8
CRi (CR with incomplete count recovery)	248 (32)	1 (33)		
No remission	181 (24)	0		
Allogeneic stem cell transplant: (*n* = 258)	256 (34)	2 (1)	0	**<0.0001**
Alive	250 (33)	14 (10)	18 (8)	**<0.0001**
Dead	516 (67)	130 (90)	195 (92)	
Survival, months (median, range)	22.5 (20–27)	8.8 (7.0–11.4)	2.2 (1.6–3.0)	**<0.0001**

Bold values indicates the variable to describe primary and secondary AML including their *p* values.

### Survival

**Fig. 1:**
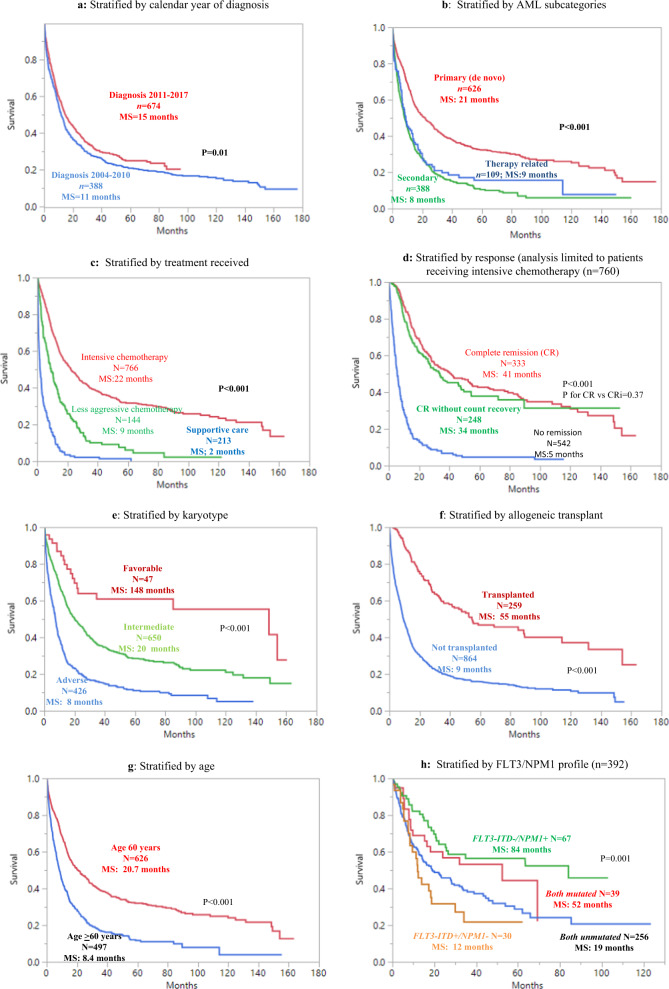
Overall survival data among 1123 consecutive adults with non-APL acute myeloid leukemia (AML). **a** Analysis stratified by calendar year of diagnosis. **b** Analysis stratified by AML subcategories. **c** Analysis stratified by treatment received. **d** Analysis stratified by response obtained (limited to patients who received induction chemotherapy (*n* = 760). MS median survival. **e** Overall survival data among 1123 consecutive adults with non-APL acute myeloid leukemia (AML) stratified by karyotype. **f** Analysis stratified by allogeneic stem cell transplant. **g** Analysis stratified by age. **h** Analysis stratified by *FLT3-ITD/NPM1* mutational status.

At the last follow-up, 841 (71%) deaths and 241 (44%) relapses were documented. The median overall survival for patients diagnosed from 2004 to 2010 and from 2011 to 2017 was 11 and 15 months, respectively; age-adjusted multivariate analysis including other risk factors confirmed improved survival in patients diagnosed in the more recent period (*p* < 0.01; Fig. 1a). Patients with primary AML displayed a better median survival of 21 months vs 8 and 9 months for secondary and therapy-related AML, respectively (*p* < 0.01; Fig. 1b). Median survivals for post-MDS (*n* = 269), post-CMML/MDS/MPN overlap (*n* = 30), and post-MPN (*n* = 89) AML, were 10, 8, and 5 months, respectively (Supplemental Fig. 1). Post-MPN AML patients had a significantly worse survival in comparison to both therapy-related AML (*p* = 0.03), and post-MDS AML (*p* = 0.03; Supplemental Fig. 1). Patients receiving intensive chemotherapy (*n* = 767) had a better survival (median 22 months) compared to those receiving less intensive chemotherapy (*n* = 144; median 9 months) or supportive care alone (*n* = 212; median 2 months; *p* < 0.01; Fig. 1c). Overall survival of patients who achieved CR (median 41 months) or CRi (median 34 months) was significantly better than those with no remission (median 5 months; *p* < 0.01); there was no statistically significant difference in survival in patients achieving CR vs CRi (*p* = 0.4; Fig. 1d).

As expected, patients with favorable karyotype (*n* = 47) displayed significantly better overall survival (median 148 months) compared to those with either intermediate (*n* = 650; median 20 months) or adverse karyotype (*n* = 426; median 8 months; Fig. 1e). Survival was generally superior in patients receiving AHSCT (median 55 months) and the value of transplant was apparent in patients achieving CR/CRi (Fig. 1f). Also, as expected, survival was better in patients aged ≤60 years (20.7 vs 8.4 months; *p* < 0.01; Fig. 1g).

*FLT3-ITD* and *NPM1* mutational information was available in 392 patients; *FLT3-ITD* and *NPM1* mutational frequencies were 18 and 27%, respectively. Figure 1e–h depicts survival stratified by mutational status and confirms superior survival in *FLT3-ITD* (−)/*NPM1* (+) (*n* = 67; median survival 84 months) and *FLT3-ITD* (+)/*NPM1* (+) cases (*n* = 39; median 52 months) compared to those with *FLT3-ITD* (+)/*NPM1* (−) (*n* = 30; median 12 months) and a double unmutated profile (*n* = 256; median 19 months; Fig. 1h; *p* < 0.01).

Multivariate analysis of pretreatment parameters evaluable in all 1123 patients identified age >60 years (hazard ratio (HR) 2.2, 1.9–2.6), adverse karyotype (HR 2.9, 1.9–4.9), intermediate-risk karyotype (HR 1.6, 1.02–2.6), post-MPN AML (HR 1.9, 1.5–2.4), and other secondary AML (HR 1.3 (1.1–1.6) as risk factors for shortened survival; the inclusion in the model of *FLT3/NPM1* mutational status in 392 informative cases confirmed the adverse prognostic effect of age >60 years (HR 1.8, 1.4–2.5), adverse karyotype (HR 3.7, 1.4–15.3), post-MPN AML (HR 2.8, 1.6–4.6), other secondary AML (HR 1.4, 1.0–1.9), and *FLT3*+*NPM1*− (HR 2.8, 1.4–5.6), *FLT3*+/*NPM*+ (HR 2.6 (1.3–5.2), and *FLT3*−*NPM1*− (HR 1.8, 1.0–3.0) profile. We have published the impact of mutations (FLT3, NPM1, and CEBPA) in non-favorable karyotype^11^. Results were unchanged when survival was censored at the time of ASCT. Two hundred and forty-one relapses were documented during the study period. Median survivals of patients with and without relapse were 23 and 154 months, respectively (*p* < 0.01); median relapse-free survival of patients with and without AHSCT was 59 and 27 months, respectively.

## Discussion

We report the largest cytogenetically annotated single-center real-world management experience of patients with non-APL AML (*n* = 1123). At diagnosis, the median age was 65 years and with male predominance (3:2) and is comparable to US census data where the median age was reported to be 68 years (https://www.cancer.org/cancer/acute-myeloid-leukemia/about.html, https://seer.cancer.gov/statfacts/html/amyl.html).

Patients with primary AML tended to have higher leukocyte count and bone marrow blast percentage and favorable or intermediate cytogenetic risk groups in comparison to secondary or therapy-related AML. These findings were previously documented in other retrospective and population-based studies^12–15^. Patients with secondary and therapy-related AML were older and this might be partly related to their history of antecedent hematological malignancy and exposure to chemo-radiation. These underlying predisposing factors may affect the bone marrow stem cells and micro-environment leading to progression of the disease to acute leukemia and further clonal evolution contributing to unfavorable cytogenetic and molecular profile.

Several studies have previously suggested that patients receiving induction chemotherapy lived longer, in particular those achieving CR^16^.

Our patients who were diagnosed in the more recent study period (2011–2017) displayed better overall survival than those diagnosed earlier (2004–2010); this may be attributed to continuing improvement in supportive care therapy, including frequent use of prophylactic antibiotics. In addition, more patients (83 vs 78%) received therapy during 2011–2017, including 78% of those who received less intensive therapy. This may be attributed to the wide availability of and physician preference to less intensive therapy.

As expected, patients with primary AML enjoyed better overall survival, compared to those with secondary or therapy-related AML. Multiple factors contribute to this observation, including younger age, better performance status, lesser likelihood of co-morbid conditions, and more favorable cytogenetic and mutation profile, associated with primary vs secondary/therapy-related AML^14^. In general, patients with primary AML have favorable host factors that enable them to tolerate more intensive chemotherapy. Our observations in this regard are similar to a previously published population-based cohort study^13^.

Regardless of other factors, patients who received intensive chemotherapy displayed a better overall survival in comparison to those receiving less intensive or no therapy. Furthermore, the current study suggested that the quality of complete remission (CR vs CRi) did not affect overall survival. In this regard, our observation is somewhat different from that of Greef et al. and Walter et al.^17,18^. Similar to multiple other previously published studies, the current study confirms the value of risk stratification based on age, eligibility for AHSCT, karyotype, and mutation profile. In the current study, unfavorable karyotype was more common in older patients and associated with poor outcome, supporting the rationale to wait for cytogenetic and molecular reports in such group of patients before initiating therapy, as previously suggested by another study^19^. Similar to other studies, *NPM1* mutation in the current study was associated with better survival both in the presence and absence of *FLT3-ITD* and the latter without *NPM1* mutation carried the worst prognosis^20^.

The prognosis and outcome for non-APL AML depends on both host factors and the biology of the leukemic blasts and their microenvironment. The individual host factors that may affect prognosis and decision with regard to choice of therapy may include age, performance status, and co-morbidity^21^. Despite the origin of myeloid blasts, whether primary or secondary, it is the genetic composition of blasts that primarily determines the prognosis and outcome to therapy^22–25^. Almost all patients with AML harbor somatic genomic mutations that are often categorized as *driver (disease initiation)* and *second hit or passenger* (responsible for disease manifestations and/or progression) mutations. Some of the driver mutations (*DNMT3A*, *TET2*, and *ASXL1* so-called ***DTA***, *IDH1/2*)^26^ are commonly identified as founding clones^27–29^, and may be found in asymptomatic elderly individuals^30,31^, rarely cause overt leukemia by themselves but may decrease the threshold for malignant transformation. Some of the passenger mutations may include signal transduction (*FLT3*, *NRAS*, *KRAS*, *C-KIT*, and *PTPN11*), *NPM1*, spliceosome (*SRSF2*, *SF3B1*, *U2AF1*, *ZRSR2*), chromatin regulation (*EZH2*, *BCOR*), or cohesin (*STAG2*) genes, and others may accelerate the progression to overt leukemia^32^. Occasionally, passenger mutations, similar to specific cytogenetic abnormalities, may help to separate de novo AML from secondary AML^33–35^. Driver mutations may serve as a substrate for relapse after chemotherapy or AHSCT.

Despite the limitation of this study because of its retrospective nature, single center, lack of molecular data in the majority, and underrepresentation of newly approved drugs, this real-life experience study could serve as a benchmark against which we may measure outcomes of newly approved therapies. Finally, we were encouraged by the improvement in survival overtime and further improvement is likely, considering the introduction of targeted therapy (e.g., *FLT3* and *IDH* inhibitors) and incorporation of Venetoclax into low-intensity chemotherapy.

## Supplementary information

Supplemental Figure 1
