# Supplementary figures and images for: Mayo Clinic experience with 1123 adults with acute myeloid leukemia
Source: Blood Cancer J. 2021 Mar 2;11(3):46. doi: 10.1038/s41408-021-00435-1 (PMC7925511; doi:10.1038/s41408-021-00435-1)

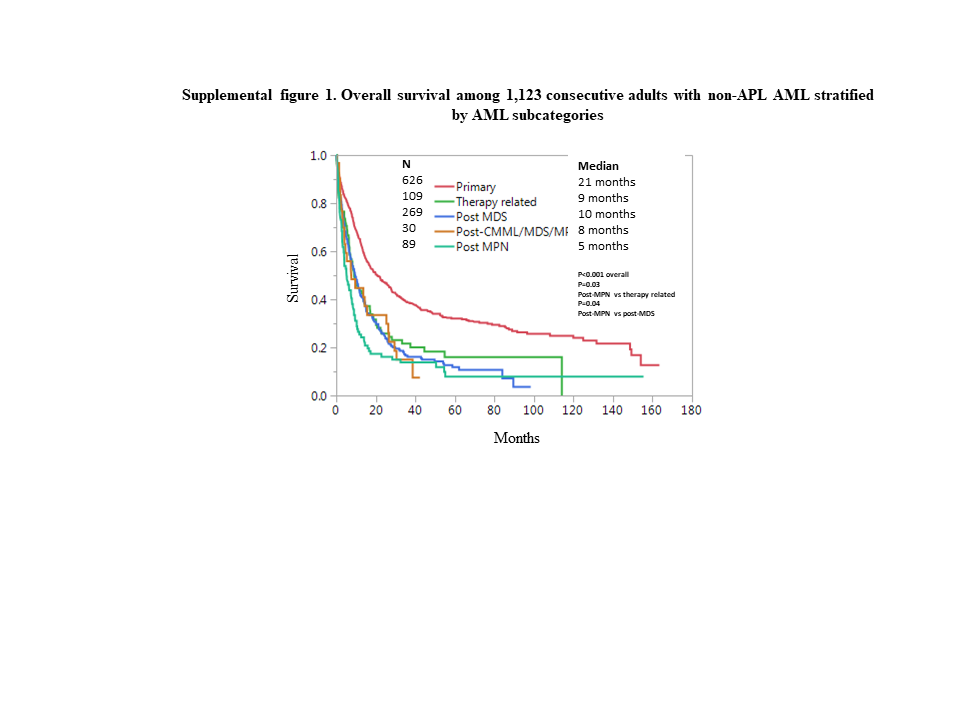

Supplement: Supplementary file 1 — Supplemental Figure 1 [file 41408_2021_435_MOESM1_ESM.tif]
